# Online medical history taking course: Opportunities and limitations in comparison to traditional bedside teaching

**DOI:** 10.3205/zma001555

**Published:** 2022-07-15

**Authors:** Silvan Lange, Nils Krüger, Maximilian Warm, Mark op den Winkel, Johanna Buechel, Johanna Huber, Orsolya Genzel-Boroviczény, Martin R. Fischer, Konstantinos Dimitriadis

**Affiliations:** 1 University Hospital, Ludwig-Maximilians-University (LMU) Munich, Institute of Medical Education, Munich, Germany; 2 University Hospital, Ludwig-Maximilians-University (LMU) Munich, Department of Internal Medicine III, Munich, Germany; 3 University Hospital, Ludwig-Maximilians-University (LMU) Munich, Department of Internal Medicine II, Munich, Germany; 4 University Hospital, Ludwig-Maximilians-University (LMU) Munich, Department of Gynecology and Obstetrics, Munich, Germany; 5 University Hospital, Ludwig-Maximilians-University (LMU) Munich, Dr. von Hauner Children's Hospital, Division of Neonatology Campus Innenstadt, Munich, Germany; 6 University Hospital, Ludwig-Maximilians-University (LMU) Munich, Department of Neurology, Munich, Germany; 7 University Hospital, Ludwig-Maximilians-University (LMU) Munich, Institute for Stroke and Dementia Research (ISD), Munich, Germany

**Keywords:** medical history taking, communication, online teaching, online training, distance education, undergraduate medical education, COVID-19

## Abstract

**Objective:**

Obtaining a systematic medical history (MH) from a patient is a core competency in medical education and plays a vital role in the diagnosis of diseases. At the Faculty of Medicine at LMU Munich, students have their first course in MH taking during their second year. Due to the COVID-19 pandemic, the traditional bedside MH taking course had to be transformed into an online course (OC). Our objectives were to implement an online MH taking course, to evaluate its feasibility and to compare the evaluation results to a historic cohort that had undertaken the traditional bedside teaching course (BTC).

**Methods:**

874 second-year students participated in the OC (BTC=827). After teaching the theoretical background via asynchronous online lectures, students participated in a practical exercise with fellow students using the video communication platform Zoom where they were able to practice taking a MH on the basis of fictitious, text-based patient cases. Students were then asked to evaluate the course through a standardized online survey with 31 questions on teaching quality and self-perceived learning success, which had also been used in previous years. The survey results were compared to the results of the historic cohort using the Mann-Whitney U test.

**Results:**

A total of n=162 students (18.5%) evaluated the OC. In the historic cohort, n=252 (30.5%) completed the survey. 85.3% of the OC respondents thought that the atmosphere during the practical exercise was productive and 83.0% greatly appreciated the flexibility in terms of time management. Moreover, they appreciated the online resources as well as having the opportunity to undertake a MH taking course during the COVID-19 pandemic. 27.7% of the respondents thought that traditional BTCs should be supplemented through more online activities in the future. With respect to the ability of independently taking a MH upon completion of the course, the OC was rated significantly lower relative to the BTC (mean OC=2.4, SD=±1.1 vs. mean BTC=1.9, SD=±1.1 (1=strongly agree; 5=strongly disagree); p<0.0001).

**Conclusion:**

OCs are a feasible format and seem to convey the theory and practical implementation in a peer-exercise format of MH taking to medical students. The theoretical background can be acquired with great flexibility. Nevertheless, the students’ self-appraisal suggested that the traditional teaching format was more effective at teaching MH taking skills. Thus, we propose a blended learning concept, combining elements of both formats. In this context, we suggest prospective, randomized trials to evaluate blended learning approaches.

## Zusammenfassung

**Zielsetzung:**

Die Erhebung einer strukturierten Anamnese (ANA) eines Patienten ist eine Kernkompetenz in der medizinischen Ausbildung und spielt eine wichtige Rolle bei der Diagnose von Krankheiten. An der Medizinischen Fakultät der LMU München belegen die Studierenden im zweiten Studienjahr ihren ersten Anamnesekurs (AK). Aufgrund der COVID-19-Pandemie musste der traditionelle AK am Krankenbett in einen Online-Kurs (OK) umgewandelt werden. Unsere Ziele bestanden darin, einen Online-AK zu implementieren, seine Durchführbarkeit zu bewerten und die Bewertungsergebnisse mit einer historischen Kohorte zu vergleichen, die den traditionellen Unterricht am Krankenbett (UK) absolviert hatte.

**Methodik:**

Am OK nahmen 874 Studierende des zweiten Studienjahres teil (UK=827). Nach Vermittlung des theoretischen Hintergrunds mittels asynchronen Online-Vorlesungen nahmen die Studierenden an einer praktischen Übung über die Videokommunikationsplattform Zoom teil, in der sie die Durchführung einer ANA anhand von fiktiven, textbasierten Patientenfällen mit ihren Kommilitonen üben konnten. Anschließend wurden die Studierenden gebeten, den Kurs mittels einer standardisierten Online-Evaluation bestehend aus 31 Fragen zur Lehrqualität und zum selbst wahrgenommenen Lernerfolg zu bewerten. Diese kam auch in den Vorjahren zum Einsatz. Die Evaluationsergebnisse wurden mit den Ergebnissen der historischen Kohorte mit Hilfe des Mann-Whitney-U-Test verglichen.

**Ergebnisse:**

Insgesamt bewerteten n=162 Studierende (18,5%) den OK. In der historischen Kohorte beantworteten n=252 (30,5%) die Evaluation. 85,3% der OK-Befragten empfanden die Atmosphäre während der praktischen Übung als produktiv und 83,0% schätzten die Flexibilität bezüglich der Zeiteinteilung sehr. Darüber hinaus schätzten sie die Online-Ressourcen sowie die Möglichkeit, während der COVID-19-Pandemie an einem ANA-Kurs teilzunehmen. 27,7% der Befragten waren der Meinung, dass traditionelle UKs zukünftig durch mehr Online-Aktivitäten ergänzt werden sollten. In Bezug auf die Fähigkeit, nach Abschluss des Kurses selbstständig eine ANA zu erheben, wurde der OK im Vergleich zum UK signifikant schlechter bewertet (Mittelwert OK=2,4, SD=±1,1 vs. Mittelwert UK=1,9, SD=±1,1 (1=trifft voll zu; 5=trifft gar nicht zu); p<0,0001).

**Fazit:**

OKs sind ein praktikables Format und scheinen die Theorie und praktische Umsetzung in einem Peer-Übungsformat der ANA-Erhebung an Medizinstudierende zu vermitteln. Der theoretische Hintergrund kann flexibel erworben werden. Die Selbsteinschätzung der Studierenden deutete darauf hin, dass das traditionelle Unterrichtsformat effektiver war, um ANA Erhebungsfähigkeiten zu vermitteln. Daher empfehlen wir ein Blended-Learning-Konzept, das Elemente beider Formate kombiniert. In diesem Zusammenhang schlagen wir prospektive, randomisierte Studien vor, um Blended-Learning-Ansätze zu evaluieren.

## 1. Introduction

### 1.1. Background

The systematic inquiry in a patient’s medical history (MH) plays a vital role in the diagnosis of diseases [1], [2]. Doctor-patient communication is also beneficial for the patient’s wellbeing [3], [4]. Therefore, medical students are often taught how to take a systematic MH early in their studies [5].

MH taking is a core competency in medical education. Some countries, such as the USA, have even classified it as an Entrustable Professional Activity (EPA), thus indicating that students should be able to obtain a complete and accurate MH in an organized fashion and demonstrate patient-centered interviewing skills [6]. This is also stated in the German National Competency-based Learning Objectives Catalogue (NKLM) [https://www.nklm.de]. As preclinical medical students have not yet acquired sufficient medical expertise in order to identify differential diagnoses, it is mainly expected of them to learn about the structure of a MH and different questioning techniques.

These skills can be taught using different teaching formats. Keifenheim et al. [7] performed a systematic review to analyze different formats. They presented several approaches. Traditional methods of teaching how to take a MH included: focus scripts [8], videotape review [9] and an online course [10]. Another format featured “learning by doing” approaches involving small group workshops including role-play and feedback [11], [12], small group workshops including simulated patients [13], [14], [15] and virtual patients [16] and small group workshops including real patients [17], [18]. Additionally, creative approaches such as improvisational theatre [19], [20] and Lego^®^ simulation [21] were described. Overall, they concluded no superiority of one specific method over the other [7].

Peer teaching appears to be equally effective as traditional teaching formats in teaching MH taking [22]. Active participation and collaboration are also essential to consider when choosing an appropriate teaching format as they help to increase students’ learning success as suggested by the ICAP model (Interactive, Constructive, Active and Passive) [23], [24].

#### 1.2. Problem

At LMU Munich, students have their first course in MH taking in the second year of their studies. Traditionally, students had in-person lectures with practical examples of taking a systematic MH followed by a bedside teaching course (BTC). This can be seen as a combination of Keifenheim et al. [7] traditional learning method combined with a “learning by doing” approach using real patients.

The COVID-19 pandemic, however, has had great implications for traditional teaching formats in medical education and many courses had to be delivered online [25], [26]. In our faculty, the BTC for second-year students was no longer permitted. Instead, an online MH taking course was designed and first applied to the second-year cohort during winter term 2020/2021.

In the past, the above-mentioned methods have been proven to be effective in teaching MH taking [11], [17], [27]. However, evidence on the effectiveness of online courses (OC) is scarce. Some authors implemented OCs that aimed at teaching different questioning styles and nonverbal communication [10]. Kyaw et al. systematic review [28] concluded that OCs may be equally effective in teaching communication skills compared to traditional learning methods. In contrast, Fink et al. [28] suggest that although cognitive load was similar, students who participated in a MH taking course with virtual patients had a reduced diagnostic accuracy as well as a reduced perceived authenticity compared to a course using standardized patients (SP) [29]. Moreover, there is a growing number of reports on “Zoom fatigue”, which may affect students’ learning success online [30], [31].

A number of universities in Germany implemented online MH taking courses during the COVID-19 pandemic with promising results. One approach at the Goethe University in Frankfurt am Main reported on successfully establishing an online course with SPs where students reported substantial learning progress in evaluations [32]. At RWTH Aachen, the digital teaching of an online communication course with SPs was rated good by 63% of students and the digital implementation was practicable [33]. Similarly, in a digital communication course using SPs at Mannheim Medical Faculty, it was observed that students were successful in training and observing conversation techniques [34]. However, none of them compared OCs and traditional BTCs with regard to effectiveness in acquiring MH taking skills. Furthermore, students who undertook the OC at LMU Munich performed different roles during the practical MH taking exercise, whereas the other studies reported on SPs who acted as patients.

#### 1.3. Objectives

The key objectives of our study were: firstly, to implement a MH taking OC for clinically inexperienced, second-year medical students; secondly, to evaluate its feasibility in a large medical faculty; thirdly, to evaluate the course with respect to acquiring competencies as perceived by students and; finally, to compare these results to results acquired in a historic cohort of students that had undertaken the traditional BTC.

## 2. Methods

### 2.1. Study design

This was a retrospective observational study. We first implemented an online MH taking course during winter term 2020/2021. Evaluation results were then compared to those of a historic cohort, which had taken part in the traditional BTC during winter term 2019/2020.

#### 2.2. Cohorts

The MH taking course is a compulsory part of the curriculum for all second-year students at LMU Munich. Therefore, the two cohorts considered in the study (OC=874, BTC=827) consisted of a large group of female and male preclinical, second-year medical students of different age and technical affinity. A demographic comparison was not possible due to data protection issues in the setting of an anonymous evaluation. However, we assume that the two cohorts were similar. The OC cohort had been taught with an in-person teaching format and an online format each for one semester during their first two semesters before undertaking the OC in their third semester. The BTC cohort was taught solely with an in-person teaching format. Neither of the two cohorts had patient contact before undertaking the MH taking course.

#### 2.3. Online course

##### 2.3.1. Learning objectives

Upon completion of the course, students were expected to be able to take a systematic MH, to name its components, as well as to show a clear understanding of the use of different questioning techniques. Students had to acquire expertise with regard to the structure and form of a MH, as well as gaining competencies in communication skills. The learning objectives were found on the online learning platform Moodle.

Based on the literature, we chose the small group workshop and role-play format for the course. Through this, students had the opportunity of taking a MH during a practical peer-exercise with two fellow students via the communication platform Zoom (Zoom Video Communications, San Jose, CA, USA). We added a feedback element as this enhances the learning experience [12].

##### 2.3.2. Learning resources

Prior to the practical exercise, students had access to a wide range of learning resources on Moodle, which they were able to use during a four-week preparation phase. This included a question template for MH taking, learning objectives, five online lectures as well as two example videos of taking a MH.

##### 2.3.3. Fictitious patient cases

When designing the OC, we had to consider that, due to the COVID-19 pandemic, there would be no real patients with whom students could practice MH taking. Therefore, we created 60 fictitious patient cases, which students used during the practical exercise. The diagnoses in the cases referred to common illnesses found in internal medicine, e.g. pneumonia. Each case was divided into the following sections: “patient details”; “history of present illness”; “past MH”; “allergies”; “family diseases”; “social history”; “travel history”; and “review of systems”. All cases were reviewed by internal medicine specialists.

##### 2.3.4. Practical history taking exercise

**Figure 1:**
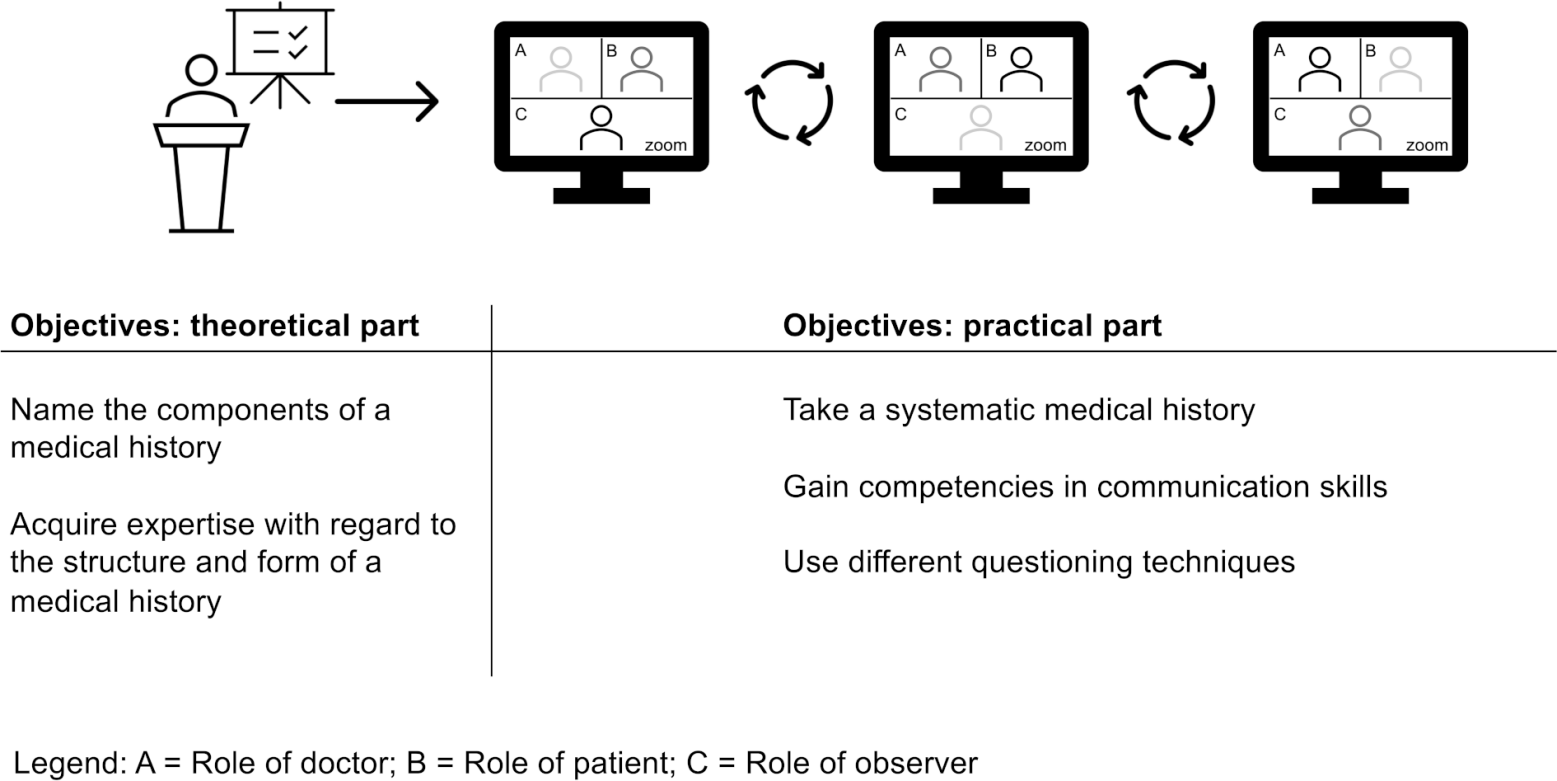
Procedure and learning objectives of the online history taking course

In order to carry out the practical exercise of taking a MH, all students were randomly allocated into groups of three. The groups were given a period of two weeks to practice taking a structured MH via Zoom. The students had approximately ten minutes to take the MH and played the roles of the “doctor”, “patient” and “observer”. Each student received a fictitious patient case at random containing all required information to play the role of the “patient”. After a student had finished taking the MH, the students swapped roles. Each interview was followed by a peer feedback session regarding the MH taking skills of the “doctor” (see figure 1 (Fig. 1)). Here, students acted as teachers using peer teaching. This allowed us to further integrate peer teaching into the curriculum using formative oral feedback [35] so that students could benefit from the course from the perspective of a teacher as well as a learner [36]. As proof of completion, students uploaded their recorded interview to Moodle.

##### 2.3.5. Technical aspects

An instruction manual for the communication platform Zoom was uploaded to Moodle; here the students carried out the practical MH taking exercise. Students had the opportunity of reporting technical problems to our email address.

#### 2.4. Traditional bedside teaching course

The MH taking course, which had been undertaken by the historic cohort in winter term 2019/2020 at LMU Munich, had the same learning objectives. The main difference consisted of the bedside teaching format. It also included seven in-person lectures with an example of taking a MH performed on a SP, followed by the practical implementation of taking MHs with inpatients carried out in groups of three on the university hospital wards.

#### 2.5. Evaluation

##### 2.5.1. Survey development

The survey was adapted to fit the needs of evaluating the OC. The survey for the OC cohort consisted of 31 questions and was divided into five sections: “organization and technology”; “course content”; “didactics and support”; “learning success”; and “overall rating”. It consisted of 19 five-point Likert-scaled questions (verbally anchored response categories 1=strongly agree; 5=strongly disagree) (see attachment 1 for complete survey), seven open-ended questions, three dichotomous questions and two three-point Likert-scaled questions (1=entirely; 3=not at all). We deliberately used an odd number of response options to allow students to reflect moderate standing to an item [37]. The survey was based upon a frequently used standard survey for assessment of teaching quality at LMU Munich (see attachment 1 ).

##### 2.5.2. Data collection

Upon completing the course, students in both cohorts were sent a link to an online survey. The participation in the survey was voluntary and had no influence on students’ grades. Furthermore, all responses were anonymous.

#### 2.6. Statistics and analysis

**Figure 2:**
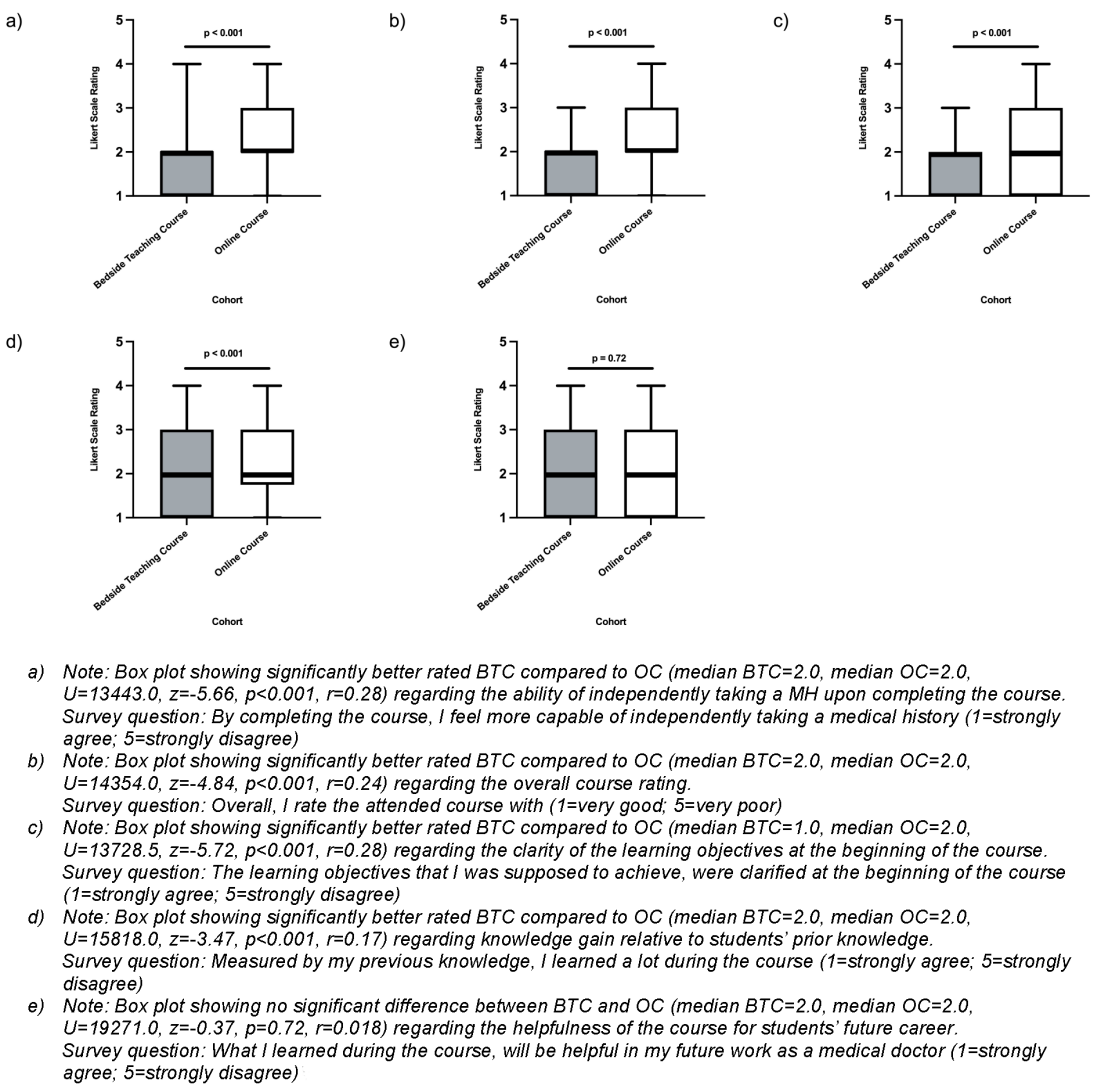
a) Medical history taking ability upon completing the online course compared to bedside teaching course b) Overall comparison of online history taking course and bedside teaching course c) Clarity of learning objectives in the online history taking course compared to the bedside teaching course d) Students’ knowledge gain relative to their prior knowledge on the topic before and after completing the online vs. bedside teaching course e) Helpfulness of the medical history taking course for students’ future work as medical doctors (bedside teaching course vs. online course)

By means of descriptive statistics, we compared five corresponding items (see figure 2 (Fig. 2)) of the two cohorts using the Mann-Whitney U test. A significance level of p<0.05 was used for all tests. Tests were carried out using SPSS Statistics (version 28.0). Open-ended questions were categorized and summarized by topic using summarizing qualitative content analysis.

#### 2.7. Ethics

The study was conducted in conformity with the Declarations of Helsinki and Geneva. The study protocol was approved by the ethical review board of the Faculty of Medicine of LMU Munich (project nr. 20-788).

## 3. Results

### 3.1. Course comparison: online vs. bedside teaching course

#### 3.1.1. Cohorts and samples

A total of n=874 second-year medical students undertook the OC in winter term 2020/2021. The survey was answered by n=162 students (response rate=18.5%). 60.8% of respondents stated that they had no prior experience in taking a MH. In the historic cohort, n=827 students participated in the BTC. The survey was answered by n=252 (30.5%).

##### 3.1.2. Quantitative analysis

With regard to the ability of students independently taking a MH upon completing the course, the BTC, based on self-perception, was rated significantly better compared to the OC (median BTC=2.0, median OC=2.0, U=13443.0, z=-5.66, p<0.001, r=0.28) (see figure 2 (Fig. 2), point a). Moreover, the overall rating of the BTC was significantly better than the OC (median BTC=2.0, median OC=2.0, U=14354.0, z=-4.84, p<0.001, r=0.24) (see figure 2 (Fig. 2), point b). The learning objectives were clarified significantly better in the BTC compared to the OC (median BTC=1.0, median OC=2.0, U=13728.5, z=-5.72, p<0.001, r=0.28) (see figure 2 (Fig. 2), point c) and, relatively to their prior knowledge on the topic, students learned significantly more in the BTC compared to the OC (median BTC=2.0, median OC=2.0, U=15818.0, z=-3.47, p<0.001, r=0.17) (see figure 2 (Fig. 2), point d). Both cohorts showed approval with respect to the statement that they learned something, which will be helpful for the future work in their career as medical doctors (median BTC=2.0, median OC=2.0, U=19271.0, z=-0.37, p=0.72, r=0.018) (see figure 2 (Fig. 2), point e). Except for the last item (non-significant difference), all items showed a significance difference of p<0.001 and the r-values were of medium effect size [38].

##### 3.1.3. Qualitative content analysis

The summary of the open-ended questions of the OC was based on a total of 239 responses. The respondents praised the online resources, the flexible time management during the practical MH taking exercise as well as the ability to practice taking a MH with fellow students before being exposed to real-life patients. For instance, one student stated: “The flexible time allocation was extremely relieving” and another replied: “I appreciated taking my first medical history with a fellow student without feeling inhibited.” The main request for the future was that MH taking should be practiced in a BTC with real-life patients. A student responded: “MH taking on a real patient can’t be replaced by a digital exercise”. However, several students welcomed the online format and are in favor of a hybrid course combining teaching formats of both OC and BTC. A student stated: “A hybrid course would be ideal.”

In the BTC, a total of 237 responses to the open-ended questions were administered. The insight into hospital wards and the ability to practice taking a MH with real-life patients were especially appreciated. One student answered: “Everyone had the opportunity of taking a patient’s MH”. There was a mixed view on the preparedness of the supervising doctors on the wards. For example, a student replied: “The supervisor was very motivated and was able to answer questions in a helpful manner”, whereas another student stated: “The doctors weren’t informed about our coming, nor about the course and the learning objectives of the course”. The organization of the course with respect to finding the right ward was criticized and there was a request for more interactive lectures.

#### 3.2. Online course feasibility and acceptance

All groups of three managed to conduct the practical MH taking exercise and to upload it to Moodle as a proof of completion. We received no negative feedback from students regarding the course implementation.

The online learning resources were used by 96.2% of respondents and the respondents indicated that the learning objectives were taught understandably (mean=1.7, SD=±0.8). 6.3% of respondents experienced technical difficulties during the practical exercise; the main problem was an unstable Internet connection. Peer feedback was considered very helpful during the practical exercise (mean=1.8, SD=±1.0). 85.3% of the respondents thought that the atmosphere during the practical exercise was productive and 83.0% greatly appreciated the flexibility in terms of time management. 27.7% of the respondents thought that traditional BTCs should be supplemented through more online activities in the future.

## 4. Discussion

Our results show that an OC, using small group workshops and role-play, is a feasible and implementable format to teach MH taking to preclinical medicine students. This was also shown in other studies [32], [33], [34]. These studies had students with different clinical experience and a smaller cohort in comparison, each consisting of approximately n=400 students.

In accordance with Ullmann-Moskovits et al. [32], our report shows that students greatly appreciate the flexibility of working from home, including time management, the example videos of taking a systematic MH, as well as the role plays during the practical exercise.

In comparison to traditional BTCs, the responses to the survey regarding the OC were rated less positively. This was particularly noticeable when looking at the overall course rating and the ability of students independently taking a MH upon completing the OC. This is surprising as the chosen OC format was assessed positively in Keifenheim et al. [7] systematic review. Moreover, the ICAP model suggests that an OC should be favorable for the students’ learning experience as it promotes constructive and collaborative learning activities within the practical exercise [23], [24]. Finally, peer teaching and group learning further improves learning success [39].

When considering why students’ learning success is greater with real-life patients compared to OCs, the literature review of Peters and Ten Cate [40] can be taken into account: students benefit from bedside teaching by gaining experience of the patient-doctor relationship, as they can directly observe patient-centered care [41]. Furthermore, experiences with real patients are essential for students in order to elaborate the structured mental models of diseases as a safe learning environment is provided [42]. In order to enhance students’ learning experience however, small sized groups and adequate space for BTCs are required [43].

Contrarily to our results, Hartmann et al. [34] proposed that students had a similar experience with online SPs compared to traditional classroom teaching. Tates et al. [44] suggested that there is no significant difference when comparing screen-to-screen and face-to-face consultations, concerning patient-related outcomes, satisfaction and relationship building. The results were also evaluated using self-perception questionnaires. However, the outcomes resulted from the change of just one variable, and they were based solely upon 48 simulated consultations, which had been conducted by fifth- and sixth-year medical students. This shows a major difference to our cohort, where the course traditionally presented a first point of clinical contact for second-year students. Therefore, it is more difficult to transfer this learning environment to OCs when taking a MH with fellow students.

Certainly, an OC has its benefits, such as the use of pre-recorded example videos of taking a systematic MH to convey the structure of taking a MH as well as offering students greater flexibility [25]. However, this course, as traditionally taught, was a highlight in the preclinical curriculum where the theory of the first two years of medical school was enhanced by a BTC with real patients. Therefore, it was not surprising that the students’ main critique point was that there was no patient contact whilst taking a MH.

The majority of students disapproved of expanding the OC portfolio. The negative feedback concerning further supplementation of online activities in the future, may be linked to a progressing “Zoom fatigue” during the COVID-19 pandemic [30].

Synthesizing the results of our study, with highly appreciated OC elements mainly during the preparation phase, together with the highlighted importance of a practical course in a real-life setting, a blended learning concept might suit perfectly. As summarized in Rowe et al. [45] systematic review, traditional teaching formats, practical exercises and computer-based tools can help students to bridge the gap between theory and practice [46]. The use of interactive OCs and augmented clinical learning leads to a better understanding of the relationship between theory and practice in real-world clinical scenarios [47]. Furthermore, a flipped classroom teaching concept may be beneficial for students’ learning success and, in some cases, might be preferred by students to traditional teaching formats [48]. The flipped classroom format can also be used as an effective tool for procedural learning. The flipped classroom approach requires students to independently acquire foundational knowledge, which is then applied during in-person seminars [49]. Objective Structured Clinical Examination (OSCE) scores for surgical clinical education were significantly higher in the blended learning group compared to the face-to-face group [50]. Although there is some evidence in literature concerning blended learning concepts to improve communication skills, data on MH taking is scarce. Gordon et al. [51] found that lectures and focused-examination training led to improved knowledge and clinical competencies in MH taking.

A blended learning approach for MH taking, consisting of online learning as well as in-person learning experiences, should be considered as an option for delivering future courses. This would allow students to acquire the theoretical background through flexible online lectures as well as practicing taking their first MH in a safe surrounding with fellow students followed by the practical implementation on wards with real patients.

### Limitations

As mentioned, no demographic data was collected during the surveys. There is no reason to assume a disparity in the two cohorts, as both consisted of a large group of preclinical, second-year medical students.

This MH taking course traditionally presented students’ first point of patient contact. The OC cohort had undergone two semesters of online teaching in comparison to the cohort which had solely in-person teaching. As neither the OC nor the BTC cohort had patient contact before undertaking the MH taking course, it can be assumed that both groups have the same starting point in terms of MH taking.

The overall response rate corresponds to typical voluntary survey response rates at LMU Munich [52], [53]. However, a stronger bias towards gender or age may be given by a response rate of 18.5% in the OC and 30.5% in the BTC.

As the BTC was held before the COVID-19 pandemic, the structure and content of the BTC survey determine the comparable items regarding the questions in the two surveys. Also, the survey only enquired students’ subjective self-perception; the use of objective measures to evaluate MH taking performance, such as using an OSCE, were not applied. This could be used to objectively test the communication competencies of medical students [54], to objectify students’ self-perception and to directly compare communication competencies of an MH taking OC to a BTC.

## 5. Conclusion

Online MH taking courses appear to be feasible to convey the theory and practical implementation in a peer-exercise format of MH taking to second-year, preclinical medical students. However, according to the students’ perception, the BTC was more effective in teaching MH taking skills. Thus, we propose a blended learning concept, combining elements of both traditional and online methods. During the COVID-19 pandemic, SPs should be considered instead of real patients. Prospective, randomized trials are required to evaluate blended learning approaches in this context. In future research, objective testing measures, such as OSCEs, should be carried out to evaluate the OC effectiveness compared to the BTC.

## Data

Data for this article are available from the Dryad Repository: [https://doi.org/10.5061/dryad.rn8pk0p9t] [55].

## Competing interests

The authors declare that they have no competing interests.

## Supplementary Material

Presentation of survey questions
