# Supplementary material for: Online medical history taking course: Opportunities and limitations in comparison to traditional bedside teaching
Source: GMS J Med Educ. 2022 Jul 15;39(3):Doc34. doi: 10.3205/zma001555 (PMC9469571; doi:10.3205/zma001555)
Supplement: Presentation of survey questions [file JME-39-34-s-001.pdf]

Attachment 1: Presentation of survey questions

**Evaluation Online History Taking Course**	**Question-Type**
**Organization and Technology**	
It was clearly communicated from the beginning how the online course format will look like.	Five-point Likert scale:<br>1=*Strongly agree*; 5=*Strongly disagree*
I was able to clarify organizational questions with the appropriate point of contact.	Five-point Likert scale:<br>1=*Strongly agree*; 5=*Strongly disagree*
Technical problems occurred before or during the teaching session.	Dichotomous:<br>*Yes*/*No*
Please, briefly describe the technical problems:	Open-ended
**Course Content**	
Have you had previous experiences in taking a patient's history?	Dichotomous:<br>*Yes*/*No*
I have used the provided online learning materials.	Three-point Likert scale:<br>1=*Entirely*; 3=*Not at all*
I have watched the instructional videos.	Three-point Likert scale:<br>1=*Entirely*; 3=*Not at all*
Why did you not, or only incompletely, watch the instructional videos?	Open-ended
By the instructional videos, the learning content was prepared understandably.	Five-point Likert scale:<br>1=*Strongly agree*; 5=*Strongly disagree*
The provided example documentation was helpful whilst documenting my own interview.	Five-point Likert scale:<br>1=*Strongly agree*; 5=*Strongly disagree*
I was able to focus on taking the patient's history in order to document the content of the interview afterwards.	Five-point Likert scale:<br>1=*Strongly agree*; 5=*Strongly disagree*
By the provided learning materials, the teaching content was prepared understandably.	Five-point Likert scale:<br>1=*Strongly agree*; 5=*Strongly disagree*
Which online materials were particularly helpful for you and why?	Open-ended
Which content is still missing? What should be improved? Please briefly describe your needs here:	Open-ended
**Didactics and Support:**	
The learning objectives that I was supposed to achieve, were clarified at the beginning of the course.*	Five-point Likert scale:<br>1=*Strongly agree*; 5=*Strongly disagree*
The info emails, accompanying the course were helpful.	Five-point Likert scale:<br>1=*Strongly agree*; 5=*Strongly disagree*
By the practical exercise via Zoom, I was able to schedule my work flexibly.	Five-point Likert scale:<br>1=*Strongly agree*; 5=*Strongly disagree*
I participated in the voluntary Q & A sessions.	Dichotomous:<br>*Yes*/*No*
The voluntary Q & A sessions contributed to my learning success.	Five-point Likert scale:<br>1=*Strongly agree*; 5=*Strongly disagree*
The working atmosphere during the practical exercise via Zoom was productive.	Five-point Likert scale:<br>1=*Strongly agree*; 5=*Strongly disagree*
The feedback from my fellow students was helpful.	Five-point Likert scale:<br>1=*Strongly agree*; 5=*Strongly disagree*
I felt well looked after during the course.	Five-point Likert scale:<br>1=*Strongly agree*; 5=*Strongly disagree*
**Learning Success:**	
By completing the course, I feel more capable of independently taking a medical history.*	Five-point Likert scale:<br>1=*Strongly agree*; 5=*Strongly disagree*

By completing the course, I feel more capable of independently documenting an interview in a structured manner.	Five-point Likert scale:<br>1=*Strongly agree*; 5=*Strongly disagree*
Measured by my previous knowledge, I learned a lot during the course.*	Five-point Likert scale:<br>1=*Strongly agree*; 5=*Strongly disagree*
What I learned during the course, will be helpful in my future work as a medical doctor.*	Five-point Likert scale:<br>1=*Strongly agree*; 5=*Strongly disagree*
I think it would be good to complement the traditional classroom teaching session with online activities in the future.	Five-point Likert scale:<br>1=*Strongly agree*; 5=*Strongly disagree*
**Overall Rating:**	
I particularly liked the following aspects of the course:*	Open-ended
From my point of view, the following aspects should be improved:*	Open-ended
How long did you take to complete the practical exercise via Zoom? Please state the duration in hours.	Open-ended
Overall, I rate the attended course with:*	Five-point Likert scale:<br>1=*Very good*; 5=Very *poor*
**Example Responses to Open-Ended Questions (Online Course):**	
“The flexible time allocation was extremely relieving.”	
“History taking on a real patient can’t be replaced by a digital exercise.”	
“I appreciated taking my first medical history with a fellow student without feeling inhibited.”	
“A hybrid course format would be ideal.”	
**Evaluation Bedside Teaching Course**	**Question-Type**
The learning objectives that I was supposed to achieve, were clarified at the beginning of the course.*	Five-point Likert scale:<br>1=*Strongly agree*; 5=*Strongly disagree*
Measured by my previous knowledge, I learned a lot during the course.*	Five-point Likert scale:<br>1=*Strongly agree*; 5=*Strongly disagree*
By completing the course, I feel more capable of independently taking a medical history.*	Five-point Likert scale:<br>1=*Strongly agree*; 5=*Strongly disagree*
What I learned during the course, will be helpful in my future work as a medical doctor.*	Five-point Likert scale:<br>1=*Strongly agree*; 5=*Strongly disagree*
Overall, I rate the attended course with:*	Five-point Likert scale:<br>1=*Very good*; 5=Very *poor*
I particularly liked the following aspects of the course:*	Open-ended
From my point of view, the following aspects should be improved:*	Open-ended
**Example Responses to Open-Ended Questions (Bedside Teaching Course):**	
“Everyone had the opportunity of taking a patient’s history.”	
“The supervisor was very motivated and was able to answer questions in a helpful manner.”	
“The doctors were not informed about our coming, nor about the course and the learning objectives of the course.”	

* Identical survey questions in online course cohort and bedside teaching course cohort used for quantitative analysis.
